# Machine Learning Culture: Cultural Membership Classification as an Exploratory Approach to Cross-Cultural Psychology

**DOI:** 10.1177/01461672251339313

**Published:** 2025-05-21

**Authors:** Kongmeng Liew, Takeshi Hamamura, Yukiko Uchida

**Affiliations:** 1 University of Canterbury, Christchurch, New Zealand; 2 Kyoto University, Kyoto, Japan; 3 Curtin University, Perth, Australia

**Keywords:** cultural difference, machine learning, cultural distance, exploratory approaches

## Abstract

Research in cultural differences generally follow top-down, theoretical approaches. This has overrepresented theories (such as individualism-collectivism) derived mainly from Western-centric observations of cultural phenomenon. We present an alternative, exploratory approach: machine learning for classifying participants’ cultural membership on international surveys. Using Wave 6 of the World Values Survey, we show that these models, paired with interpretable machine learning methods (relative variable importance and partial dependence plots), can represent magnitudes of differences between any two countries while simultaneously identifying strongly differing predictors. Analysis 1 constructs indices of cultural distance centered on USA and China, replicating previous research that used alternative methods of distance computations. Analysis 2 zooms in on USA–China, USA–Japan, and Japan–China differences, demonstrating the effectiveness of the method in both uncovering consistently known areas of cultural difference, and identifying novel dimensions for further research. Accordingly, this approach appears to be particularly effective in cultural comparisons that are traditionally overlooked.

Generations of researchers have argued for the importance of cross-cultural analyses in psychological research (Bond, 1988; Henrich et al., 2010; Markus & Kitayama, 1991; Triandis, 1995). A criticism that populations predominant in psychology research are Western, Educated, Industrialized, Rich, and Democratic (WEIRD; Henrich et al., 2010; Henrich, 2020) invigorates this point. Perhaps the most important concern is the possibility that the dominant method of sampling in the field might have led to the development of an ungeneralizable view of human psyches (Henrich et al., 2010). Cross-cultural analyses are invaluable for overcoming this problem. However, cross-cultural research in psychology has traditionally followed top-down, theory-driven approaches. For example, many cross-cultural analyses have followed the theory of individualism-collectivism (Hofstede, 2011; Triandis, 1995), independence–interdependence (Markus & Kitayama, 1991) or analytic-holistic cognition (Nisbett et al., 2001). These theories typically originate from intimate observations of East Asian societies in comparison to Western societies, with subsequent developments applying these theories to other cultural contexts, for example documenting differences in analytic and holistic thinking styles in herding/farming communities in Turkey (Uskul et al., 2008). Thus, many currently available models of culture reflect views of culture that are salient to researchers in WEIRD societies, creating a bias in cultural research that skew toward Western theorizations. Essentially, these highlight the problem of ethnocentricity in culture psychological research (Triandis, 1990), where researchers’ (often implicit) cultural attitudes and norms are often projected onto the research design.

This is classically manifested in research on happiness and well-being. The notion that individuals aim to maximize happiness is typically seen as universal, and this informs global measures like the Organisation for Economic Co-operation and Development (OECD) Better Life Index^1^ that rank countries in their subjective happiness and life satisfaction. However, research has emerged to show this to be a distinctly WEIRD (specifically, Northwestern European) ideal (see Krys et al., 2024). Moreover, despite efforts to reduce the effects of ethnocentric biases, commonly used measures of culture like individualism–collectivism and tightness–looseness (Gelfand et al., 2011) show strong correlations with cultural distance from the USA, but not China (Muthukrishna et al., 2020). This is arguably evidence of a Western-centric bias toward conceptualizations of cultural differences and reflects the tendency in the field to under-represent views of culture that may be salient in non-WEIRD societies.

This is not inherently problematic as these theoretical frameworks nevertheless provide strong foundations by which to understand how people differ around the world in their thoughts, values, and attitudes. However, what we argue is that the field lacks systematic, interpretable methods that prioritize the discovery of cultural effects in a theory-blind, bottom-up manner. Existing exploratory methods, like Exploratory Factor Analyses, provide statistical methods for clustering variables or individuals, for a conflation of various conceptual dimensions that cultures may differ on. But, they do not explicitly show how cultures are directly different from each other. For example, individualism-collectivism may be identified as a dimension by which cultures differ, and countries like the USA and Japan may differ in individualism-collectivism. However, this does not show that individualism-collectivism is the *most appropriate* dimension for differentiating between the USA and Japan.

Conceptually, bottom-up differentiation of cultures may be similar to quantifying the relative difference (or “distance”) between cultures. Muthukrishna et al.’s (2020) cultural fixation measure is a data-driven tool to derive magnitudes of cultural differences. At its core, for cross-cultural surveys, it measures the ratio of the between-culture (group) variance ( $\sigma_{g}^{2}$ ) to the total variance ( $\sigma_{T}^{2}$ ). For example, in continuous data, they first define the total variance of a sample as:

$$\sigma_{T}^{2}=\;\frac{\sum_{i=1}^{s}\sum_{j=1\;}^{n_{i}}{\left(x_{ij}-\bar{x}\right)}^{2}}{N-1}$$

where ( j ) refers to individuals nested in cultures ( i ) with a (culture) sample size ( s ) for a total sample size of N . Accordingly, cultural fixation can then be defined as:

$$\mathrm{cultural}\;\mathrm{fixation}\;=\;\frac{\sigma_{g}^{2}}{\sigma_{T}^{2}}.$$

This quantifies the relative similarity (and difference) between two cultures based on the similarity of responses within a common dataset for a given set of items. As opposed to traditional statistical comparisons that rely on comparisons of means, cultural fixation accounts for the distribution of the variances across the entire set of items in quantifying the approximate distance between these two cultures. When extrapolated iteratively across all possible dyadic combinations of data, these can inform cultural fixation indices (CFI). Using data from 80 countries (from the World Values Survey [WVS]), they centered this method on contrasting target cultures, the USA and China, to derive indices of cultural differences (distances) of every country in the WVS relative to the USA and China, in constructing the CFI.

However, this method of measuring magnitudes of cultural distances alone does not explain *why* these cultures are different. When tackling Western bias in the field, data-driven approaches should also be able to provide some explorative insight into the main drivers of these cultural differences. In other words, in quantifying how different two cultures are, we need to also identify the underlying variables that best account for this difference. For example, Japan and China are two countries are often juxtaposed against the USA in cross-cultural comparisons, but little is known on how they differ from each other. Existing theories are not sufficient for differentiating between these two countries: Japan is both more collectivistic (Oetzel et al., 2001) and less collectivistic (Oyserman et al., 2002) than China. With the absence of suitable theory, a data-driven methodology could help to establish (1) how different they are from each other (vis-à-vis the USA) and (2) why they’re different: What are the most important reasons or variables that account for these relative cultural differences?

To achieve this, we propose the use of cultural membership as the outcome variable in interpretable machine learning models as detailed below. Not only can the accuracy of machine learning models function as estimators of the magnitude of cultural difference, machine learning models, while being suited for large sample sizes and large amounts of predictor variables, are often capable of providing variable inference. Fundamentally, this method would allow for a hypothesis-blind exploration of the magnitude of differences between two cultures, while uncovering where these differences are. We propose that this method would be best for generating explorative, data-driven insight into how cultures differ, especially in the absence of strong theoretical foundations.

## The Methodology: A Computational Approach to Cultural Membership Classification

The predominant analytical approach in psychology is to develop the best explanation of the phenomenon of interest, often through theory-driven analyses of mechanistic processes. With a data-driven machine learning approach, however, the goal is to “learn” from existing data to accurately predict (or forecast) a phenomenon of interest in new datasets (Yarkoni & Westfall, 2017). This alternative, *computational* approach, is increasingly used in psychological research (e.g., Kosinski & Behrend, 2017; Sheetal et al., 2020).

A computational approach can help illuminate cultural influences on psychological processes that were previously overlooked. With a top-down approach, most prior studies on culture focused, rightly, on a small number of theory-relevant variables, administering a carefully devised experiment or questionnaire and testing the hypothesized relationship among the variables. A data-driven approach, in contrast, would seek a model that best fits data collected from different cultural groups, regardless of the shape or structure of the data.

In examining cultural differences, we propose the use of “culture” as the outcome of interest. Consequently, a machine learning model trained to classify survey participants from two different cultures can then “learn” differences in responses between them. In cases where the variables and data are similar (such as a survey conducted on comparable demographics in two countries), such classification of cultural membership may elucidate the structure and magnitude of difference between these two cultures. Potentially, this allows for comparisons and evaluations of cultural differences in light of similar classification models with different cultures, for an efficient method to compare differences between cultures. This would allow for better integration with existing theory or even contribute to theory formation.

In considering the generalizability of computational models for exploratory cultural research, two issues must be addressed to evaluate its utility. First, the issue of *prediction*: Is it possible to train a model to accurately classify participants’ cultural membership (e.g., nationality), and if so, the issue of *interpretability*: Can such a model contribute any insight toward our understanding of cross-cultural differences? For *prediction*, a successful model that accurately classifies participants’ cultural membership based on their item responses beyond chance level indicates the presence of systematic differences between target cultures. This can be useful in examining the patterns of cultural differences, and may even uncover disparities from comparisons of traditionally “homogeneous” cultures. For *interpretability*, a common criticism of machine learning models is that they are “blackbox-ed” and impossible to interpret; even a high-accuracy model may be useless for psychologists if the reasons behind the successful predictions are inaccessible. Our solution is to utilize existing techniques in the literature that are designed to enhance model interpretability, some of which will be introduced below. If applied on a selection of theoretically relevant variables, these allow for model interpretations that can be easily understood. In this paper, we introduce dyadic culture classification models (CCMs) for cultural membership classification: Interpretable machine learning models trained to distinguish between participants from two cultures.

### Classification Accuracy as an Approximation of Cultural Difference

Our principal argument is that machine learning is able to uncover relative magnitudes of cultural differences in any two cultures based on the predictive power of a trained model. The basis of this proposed approach lies in the use of two-fold cross-validation (or the validation set approach; James et al., 2013), as a foundational tool in machine learning. This involves randomly splitting a dataset into two folds (parts), one for model building (the training set) and one for model evaluation (the holdout/test set). Traditionally, this assesses the model’s tendency to overfit: When the model possesses a strong bias toward the dataset it was developed on, with little generalizability to other datasets.

In evaluating the accuracy of a model, the model developed on the training set is used to generate a set of predicted outcomes, which are then compared against actual values in the holdout set. A strong match between both values indicates that the trained computational model has high validity or prediction accuracy. It is typically computed as the ratio of correct classifications (true positive and true negative) to all classifications made by the model:^2^

(1) $$\mathrm{Classification}\;\mathrm{Accuracy}\;=\;\frac{\mathrm{Correct}}{\mathrm{Total}}=\frac{\mathrm{True}\;\mathrm{Positive}\;+\;\mathrm{True}\;\mathrm{Negative}}{\mathrm{True}\;\mathrm{Positive}\;+\;\mathrm{True}\;\mathrm{Negative}\;+\;\mathrm{False}\;\mathrm{Positive}\;+\;\mathrm{False}\;\mathrm{Negative}}$$

For our purposes, we posit that if a model is trained to classify if participants come from one culture or another (binary classification), the resultant accuracy of the model when applied to the test set would be indicative of whether the machine can detect differences between these cultures. A high accuracy would mean that differences between cultures are sufficiently large for the model to detect consistently differing statistical patterns in predictor variables between the two cultures, such that it would easily and accurately predict (classify) participants’ cultural memberships based on these patterns. These cultural differences would also be generalizable across the training and holdout sets. Conversely, if a model is barely able to classify participants’ cultural memberships accurately beyond chance level, it would be constantly making misclassifications, as no recognizable pattern of cultural difference exists. If response biases are controlled for, missing values are properly handled, and group sample sizes are balanced, a cultural model’s classification accuracy in predicting participants’ cultural membership in the test set would thus be indicative of the magnitude of difference between these cultures.^3^

### Model Interpretation

While predictive models in machine learning are not typically designed for statistical hypothesis testing and inference, they are well-suited for exploratory analyses of large and messy datasets given the complexity of high dimensional parameters (many predictor variables) and statistical noise that such models are designed to navigate. This is because effects found in predictive models might potentially be more generalizable (less likely to overfit) and more replicable, external and sample biases notwithstanding. Moreover, models are not monolithically black boxes, and some models are capable of inference—identifying correlational relationships between predictor variables and outcome variables. In decision-tree models, such as Gradient Boosted Decision Trees (GBDTs; Friedman, 2001) used in this article, interpretation can be done through the “relative variable importance” (RVI) statistic. For GBDT, RVI is based on the frequency that a given variable is segmented in each iteration of a tree in reducing the overall residuals (Friedman, 2001). A higher RVI indicates that the variable is more instrumental in improving model accuracy. The interpretative power of RVIs is robust enough to uncover robust predictor effects, that should also appear alongside visualization methods, such as Partial Dependence Plots (PDPs). PDPs can be used to visualize relationships between the model’s predicted values and individual predictors (Friedman, 2001; Greenwell et al., 2018, see Pargent et al., 2023 for a tutorial). This focuses on the relationship between a target predictor variable and the outcome variable (or logit probability in a binomial model) while keeping all other variables constant.

As such, these computational tools offer means of model interpretation, while increasing robustness and generalizability (such as through cross-validation). Considering the needs of cross-cultural psychologists, we argue that these approaches are adept for big data explorations, allowing us to make better inferences on the strength and nature of relationships within the data, which aids in the conceptualization of novel theories and hypotheses. The strength of such approaches comes through particularly in research areas that have little or no existing literature. For example, little is known about how non-WEIRD societies might differ in their psychological tendencies. Cross-cultural comparisons between non-Western societies, like Brazil and Japan, are still relatively uncommon (De Almeida & Uchida, 2018; Salvador et al., 2026). The culture classification approach proposed here may be a method to jumpstart such endeavors.

### The Present Research

We introduce the method of culture classification through two analyses applied to the WVS dataset. For Analysis 1, we iteratively compared the USA and China against every other country in the dataset. This was to generate two indices of cultural distance relative to the USA and China based on our classification accuracy method so that they could be compared against Muthukrishna et al’s (2020) CFI-based indices of cultural distance centered on the USA and China; we reproduce their variable recoding and data cleaning from Wave 6, to test if our accuracy-based method is consistent with their CFI-based indices. Additionally, we show how the method’s use of RVIs can illuminate each dyadic cultural comparison by highlighting the domains of cultural attributes that set the USA and China apart from the rest of the world. For Analysis 2, we zoom in on 10 items on values used in Wave 6, as we think these items afford more generative interpretations of cultural differences in psychological research.

## Analysis 1

### Methods

#### Data

We used data from Wave 6 of the WVS (*N*_respondents_ = 79700, *N*_countries_ = 60 after listwise exclusion of missing data; Males = 38,235, Females = 41,465, Others/Rather not say = 70; mean age = 41.4, *SD* = 16.3, refer to our Supplemental material for detailed country-level breakdowns). The WVS comprises representative samples from countries around the world responding to questions that pertain to a wide range of individual values, such as their endorsement of tolerance to certain groups (e.g., “*when jobs are scarce, men should have more rights to jobs than women*”), personal religiosity (e.g., “*do you believe in God?*”), political stances (e.g., “*How would you place your view on this [political left to right] scale?*”), identity (“*I see myself as part of my local community*”), and subjective well-being (“*all things considered, how satisfied are you with your life as a whole these days?*”), etc. (Inglehart et al., 2014). These spanned a total of 189 relevant items as predictor variables in the dataset.

Fundamentally, by training CCMs—machine learning models trained to classify participants as belonging to one of two countries (as registered in the WVS)—we can gain insight into the specific differences between these two cultures from the items measured in the WVS. For each dyadic comparison, the dataset was subset to include only data from the two countries, from variables where 70% or more of the data were present in each country. Data were also balanced through randomized down-sampling of the larger country to match the sample size of the smaller country. For Analysis 1, this down-sampling process was repeated separately for each dyadic comparison. We examine the validity of our CCMs by correlating collated classification accuracy scores with cultural fixation (CFI) scores centered on USA and China from Muthukrishna et al. (2020), as well as Hofstede’s cultural dimension scores (Power Distance, Individualism, Uncertainty Avoidance, Masculinity, Indulgence, Long-Term Orientation),^4^ relational mobility (Thomson et al., 2018), Long-Term Orientation and Emancipation, (Minkov & Kaasa, 2021), and tightness–looseness (Gelfand et al., 2021; Uz, 2015). Finally, we note that while Muthukrishna et al. (2020) assimilated both Waves 5 and 6 of the WVS into one consolidated index, we only used data from Wave 6 to maximize consistency in variables.

#### Data Handling and Analysis

In Analysis 1, our objective was to compare the external validity of our method against the CFIs. As such, our preprocessing method followed Muthukrishna et al.’s (2020) pipeline for variable selection and recoding. This often involved condensing ordinal variables to broad levels indicative of general direction but not extent (e.g., strongly disagree and disagree may be recoded to simply “negative,” neutral would still be “neutral,” and strongly agree and agree may be recoded to simply “positive” resulting in a 3-point ordinal scale).^5^

GBDTs were trained to classify if a respondent belonged to one of two countries, based on as many items as were tested in both countries that had a maximum of 30% missing data per country. This was out of a potential total of 189 applicable items from the WVS. For each dyadic model, the extracted data were randomly split into a training and holdout set, along a 3:1 ratio. For the training set, a 10-fold cross-validation was used to determine the optimal hyperparameters for analysis (i.e., iteration, tree depth, and penalty function). Each GBDT model was fitted to the respective holdout sets to compute accuracy. To assess accuracy, we used the percentage of correct classifications in the holdout set (classification accuracy). Additionally, we report RVIs to interpret variables that contributed toward accurate discrimination of cultural membership. All GBDT models were conducted in R, using the “caret” wrapper (Kuhn, 2008) on the “gbm” package (Greenwell et al., 2022). As we compute dyadic CCMs across all possible pairings iteratively, full results are reported in our online Supplemental material, and only correlation results and aggregated statistics are reported here.

#### Results

Across 59 comparisons (excluding the target country), accuracy scores were high for both the dyadic cultural membership (CCM) classifications against the USA (USDist: Mean = 0.955, *SD* = 0.024, min = 0.898, max = 0.993) and dyadic cultural membership classifications (CCM) against China (CNDist: Mean = 0.978, *SD* = 0.015, min = 0.932, max = 1.00). The cultures closest to the USA on the USDist index included New Zealand, Singapore, Poland, and Peru, while the cultures most distant to the USA included Haiti, Egypt, India, and Rwanda. The cultures closest to China on the CNDist index included Hong Kong, Taiwan, Spain, and Sweden, while the cultures most distant to China included Malaysia, Ghana, Qatar, and Haiti.

**Figure 1.**
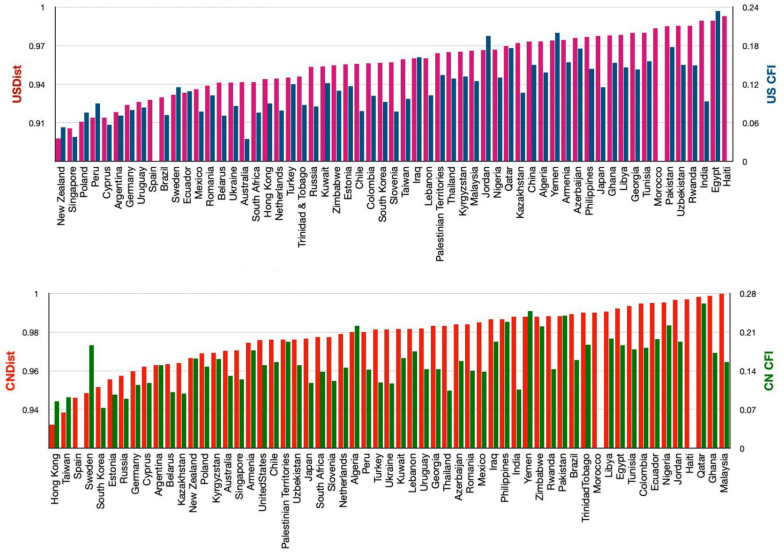
Accuracy-based distance scores and fixation indices that estimate magnitudes of differences for each country relative to the USA (top) and China (bottom).

The USDist was highly correlated with the USA CFI, *r* = .76, 95% CI [0.85, 0.62], *p <* .001, and CNDist was moderately correlated with the Chinese CFI, *r* = .63, 95% CI [0.77, 0.44], *p <* .001. These correlations demonstrate the validity of the accuracy-based distance indices for estimating cultural distances, particularly against the USA (see Figure 1). Cultures that are more distant from the USA were more accurately classified by the CCMs, supporting our assumption that the accuracy of the CCM corresponds to cultural distance between two countries.

Next, we examined external correlations to gauge the external validity of the USDist and CNDist indices. The USDist was significantly and negatively correlated with relational mobility, Hofstede’s Individualism and Indulgence, and positively correlated with Hofstede’s Power Distance. The CNDist was significantly and negatively correlated with Hofstede’s Long-Term Orientation and positively correlated with Hofstede’s Power Distance (Table 1). The USDist was also significantly and positively correlated with tightness–looseness. In short, these results are similar to what Muthkrishna and colleagues reported in relation to their CFI; the USDist was more predictive of known cultural dimensions than the CNDist index, providing additional proof of external validity of accuracy from CCMs as a measure of cultural distance.

**Table 1.** Correlations Between USDist, CNDist, and External Scales: Muthukrishna et al.’s (2020) U.S. and China Fixation Indices (CFIs), Hofstede’s Cultural Dimension Scores (Power Distance, Individualism, Uncertainty Avoidance, Masculinity, Indulgence, Long-Term Orientation), Relational Mobility (Thomson et al., 2018), Tightness-Looseness (Gelfand et al., 2021; Domain-General: Uz, 2015), and Long-Term Orientation and Emancipation (Minkov & Kaasa, 2021). Significant Effects in Bold.

Scale	*US* Dist				*CN* Dist			
	*r*	95% CI		*p*	*r*	95% CI		*p*
		Lower	Upper			Lower	Upper	
US CFI	**.76**	**0.62**	**0.85**	**<.001**	**.43**	**0.20**	**0.62**	**<.001**
CN CFI	**.29**	**0.03**	**0.52**	**.032**	**.63**	**0.44**	**0.77**	**<.001**
Relational mobility	**−.52**	**−0.75**	**−0.18**	**.005**	.03	−0.35	0.40	.872
Hofstede’s power distance	**.42**	**0.17**	**0.62**	**.002**	**.40**	**0.14**	**0.61**	**.003**
Hofstede’s individualism	**−.31**	**−0.54**	**−0.03**	**.029**	−.26	−0.50	0.02	.065
Hofstede’s masculinity	.11	−0.17	0.38	.440	.22	−0.06	0.47	.115
Hofstede’s uncertainty avoidance	.003	−0.27	0.28	.982	.05	−0.23	0.32	.714
Hofstede’s long-term orientation	−.05	−0.33	0.23	.720	**−.62**	**−0.77**	**−0.41**	**<.001**
Hofstede’s indulgence	**−.35**	**−0.57**	**−0.08**	**.012**	.13	−0.15	0.39	.368
Minkov and Kaasa’s long-term orientation	.109	−0.21	0.41	.511	**−.43**	**−0.65**	**−0.13**	**.007**
Emancipation	**−.51**	**−0.69**	**−0.28**	**<.001**	**−.45**	**−0.65**	**−0.29**	**<.001**
Tightness (looseness)	**.55**	**0.24**	**0.76**	**.001**	.29	−0.07	0.59	.113
Tightness (looseness; domain-general)	−.34	−0.63	0.04	.075	−.14	−0.49	0.24	.456

Next, for each dyadic classification, we noted the top two RVIs from among the predictor variables and collated them across all iterations. This allowed us to identify which variables of importance were repeatedly selected as the “most important variables” for USDist and CNDist. For dyadic models involving the USA, V131 “Political action: Signing a petition” was selected as the most important variable in 50.8% of dyadic comparisons, followed distantly by V191 “Believe in hell,” and V205 “Justifiable: Homosexuality,” both with 6.8% of models. For the second most important variable, V75 “Job scarce: Men should have more right to a job than a women” was the most frequent with 22.0% of models, followed by V131 again (13.6%), and V139 “Private versus state ownership of business” (11.9%).

For dyadic models involving China, V188 “Believe in: God” was selected as the most important variable in 32.2% of models, followed by V6 “Important in life: Religion” (27.1%), and V182 “How often do you attend religious services” (10.2%). For the second most important variable, V188 “Believe in: God” again was the most frequent with 15.3% of models, followed by V182 “How often do you attend religious services” and V6 “Important in life: Religion” again, both with 11.9% of models. Full reports for model RVIs for each cultural model (dyadic classification) are in our Supplemental material.

#### Discussion

Our machine learned classification of cultural membership correlates significantly and strongly with the measures of cultural distance (i.e., CFIs), which is, to our knowledge, the only other known method of computationally deriving indices of magnitudes of cultural differences from the WVS. As with the CFIs, our U.S. index was significantly correlated with more measures of culture than the Chinese index. For example, relational mobility was correlated with the U.S. index but not with the Chinese index, and measures such as individualism, power distance, and tightness-looseness were significantly correlated with the U.S. but not with the China index, replicating some of the correlations identified with the CFIs. These findings suggest that influential dimensions of culture have been calibrated more to experiences of Americans and Western populations that are contrasted to experiences of populations elsewhere; that the theory itself has been structured in a way that emphasizes the differentiation between the USA and other countries.

In focusing on the specific items highlighted by the RVIs in marking how the USA and China were different from most other countries, we note that, by a large margin, the USA was marked by political action, specifically experience in signing a petition. Between the first and second-placed RVIs for all CCMs, this was identified in a majority (64.2%) of cultural comparisons. By contrast, other items were markedly more subdued. “Belief in hell” tended to be prominent only when comparing with other WEIRD countries (e.g., Germany, Sweden, New Zealand, the Netherlands), and “homosexuality as justifiable” also tended to be prominent when contrasting against largely religious societies (e.g., Georgia, Trinidad and Tobago, Zimbabwe, and Uzbekistan).^6^ Similarly, issues about gender and equal rights at work were also more prominent in comparisons with conservative-traditional countries (e.g., Algeria, Egypt, Libya, Malaysia),^7^ and issues about private versus state ownership of companies were prominent in comparisons with formerly communist countries (e.g., Russia, Kazakhstan, Poland, Ukraine).

For Chinese distances, we noted that the three identified items on religiosity dominated across CCMs for the top two variables of importance, and in the topmost RVI alone, these three items spread across 69.5% of dyadic models. This is consistent with past research on Chinese religious agnosticism. A 2010 Pew Research demographic study on world religions found that 700 million Chinese (52.2%) reported having no religious affiliation, comprising 62.7% of the global population of religiously unaffiliated individuals (Pew Research Center, 2012). Of our Chinese-focused CCMs, only comparisons with Australia (24.2%), Estonia (59.6%), Germany (24.7%), Hong Kong (56.1%), Japan (57.0%), South Korea (46.4%), Sweden (27.0%), and India (<0.1%) did not have one of these three items (belief in God, religiosity, and religious activity participation) highlighted as the top two variables of importance. Apart from India, these countries have high rates of religious agnostic individuals in their populations (percentages in brackets; Pew Research Center, 2012).

One major benefit of RVIs in the construction of this index is that it provides a window as to how and why some countries are more distant from others. For example, China appears to be culturally closer to Spain, Sweden, and Germany, than it is to Malaysia. At first glance, this appears counterintuitive—China is geographically closer to Malaysia, and ethnically more similar to Malaysia (where 23% of residents are ethnically Chinese), as compared to Western Europe. Yet, why is Malaysia the most distant country from China? The RVIs provide a possible explanation. China is by far one of the most religiously agnostic country in the dataset, and Malaysia has been seeing a massive resurgence of religiosity in society since the mid-20th century (Ebrahimi & Yusoff, 2020) and has very low rates of religious agnosticism (0.7%). It is likely that the differences in attitudes toward religion were sufficiently large enough to influence cultural membership classification, as machine learning appears to “focus” on important variables in classification (see section “Limitations”). We note that other countries that are culturally distant from China in this dataset are also highly religious societies (Haiti, Ghana, Qatar), and while it may not capture the entire picture, RVIs offer at minimum a glimpse into potential underlying explanations. Overall, RVIs showed the items which “contributed” the most to cultural membership classifications. These items allow for across-the-board comparisons to identify specific areas where cultures differ, and in our USA and China distance measures, these appeared to be consistent with the literature. However, while RVIs “show where to look,” they do not show the direction of difference, and our inferences in this section were based on contextual knowledge and previous research. For that we need to run the computationally more intensive PDPs, so we zoom in on cultural differences in the USA, Japan, and China as a case study in Analysis 2.

## Analysis 2

We noticed in Analysis 1 that including a wide breadth of items led to findings that may not be most applicable to psychology researchers. For example, the RVIs highlighted variables pertaining to religion (or lack of) in China and political action in the USA, but a psychologist specializing in interpersonal relations might find it difficult to weave these results into their research. One practice has been to select predictors for machine learning models based on a spread of relevant variables from the literature (e.g., Lou et al., 2022). Accordingly, while we advocate for a hypothesis-free, bottom-up exploration of data through our computational approach, we recognize that many researchers in psychology may find more utility in bringing *some* foundational theoretical relevance to selecting variables of interest. In this section, we demonstrate this process in using WVS items on values and examine the extent to which different values contribute to the classification of cultural membership, focusing closely on the USA–Japan and USA–China comparisons. With the abundance of cross-cultural research involving these dyads in East–West comparisons, our aim is to demonstrate the usefulness of our approach, particularly the validity of RVIs and PDPs in interpreting cultural difference. Following which, we use the same approach to Japan–China comparison, a dyadic pairing that is often conflated to represent a homogeneous “East Asian Confucianism.” Our aim is to interpret the results to develop an understanding of Japan-China cultural difference. We also report the results of logistic regression models: For each dyadic comparison, with respondents’ countries as the outcome variable, we conducted two logistic regression models (one full model with all Schwartz values, one reduced model with only the top three RVI values) as points of comparison with our CCMs.

### Methods

We focused our demonstration on 10 WVS items on values, as these form a well-studied theoretical framework on values (e.g., Schwartz, 2012) to evaluate our results. These items were designed to assess values that encompass people’s trans-situational goals that serve as their guiding principles (see Table 2; Schwartz, 2012), and were from the same Wave 6 of the WVS.

**Table 2.** WVS Value Items and Relative Variable Importance (RVI) List for Each of the Three Dyadic Models.

Items	Label	Value assessed	RVI		
			USA–Japan	USA–China	China–Japan
V70: “It is important to this person to think up new ideas and be creative; to do things one’s own way”	Self-direction	To be creative	4.1	12.7	10.3
V71: “It is important to this person to be rich; to have a lot of money and expensive things”	Power	To be rich	10.4	40.4	28.6
V72: “Living in secure surroundings is important to this person; to avoid anything that might be dangerous”	Security	To be safe	3.4	4.7	3.7
V73: “It is important to this person to have a good time; to ‘spoil’ oneself”	Hedonism	To have a good time	16.6	5.4	16.0
V74: “It is important to this person to do something for the good of society”	Benevolence	To do good for society	13.9	3.4	15.2
V75: “Being very successful is important to this person; to have people recognize one’s achievements”	Achievement	To be successful and recognized	5.8	6.8	6.9
V76: “Adventure and taking risks are important to this person; to have an exciting life”	Stimulation	To be adventurous	11.8	6.9	5.8
V77 “It is important to this person to always behave properly; to avoid doing anything people would say is wrong”	Conformity	To behave properly	7.2	6.1	5.3
V78: “Looking after the environment is important to this person; to care for nature and save life resources”	Universalism	To care for the environment	14.4	9.3	4.2
V79: “Tradition is important to this person; to follow the customs handed down by one’s religion or family”	Traditionalism	To be traditional and religious	12.4	4.2	12.9

While we used the same GBDT models for Analysis 2, we limited our set of predictor variables to these 10 items. Unlike Analysis 1, which compressed ordinal responses into valences, data were first normalized and centered around the participant mean to reduce effects arising from response biases between cultures. These transformed values were also used for the logistic regressions.

### Results

#### Machine Learning and Cultural Membership Classification: USA, Japan, and China

**Figure 2.**
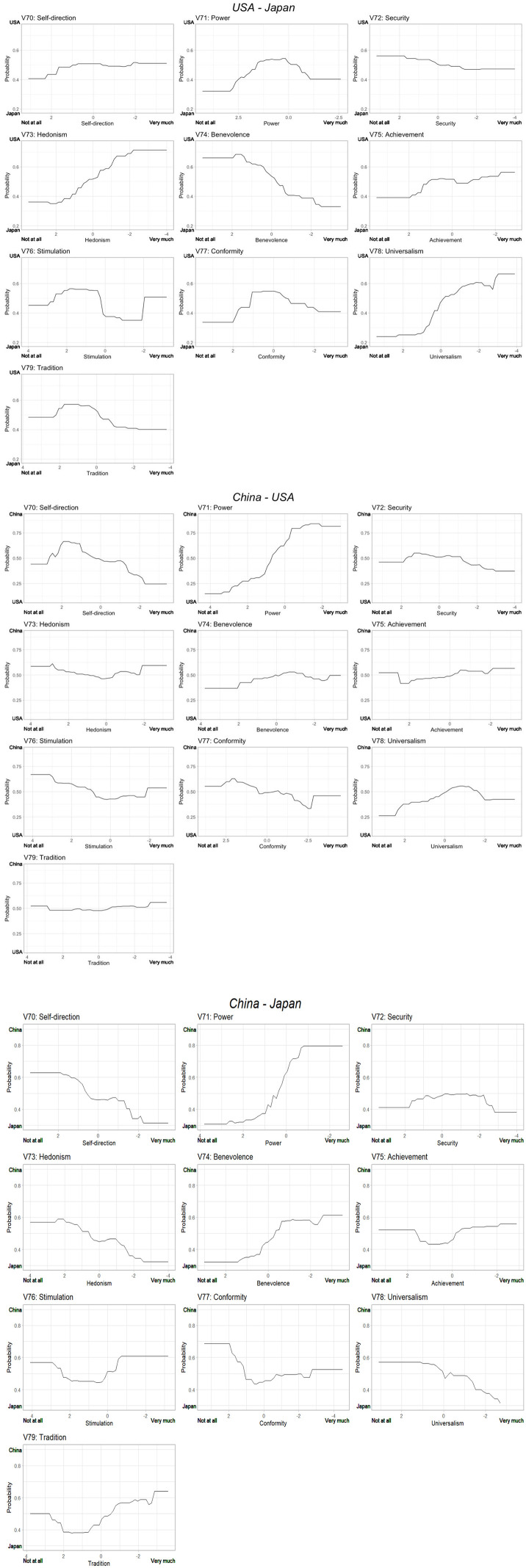
Partial dependence plots (PDP) indicate the relationship between each of 10 values and nationality from partial analysis. The top panel is for the comparison between USA and Japan, the middle panel for China and USA, and the bottom panel for USA and Japan. Results for V71, for example, show that stronger endorsement of this value was more predictive of a participant being Chinese than Japanese (bottom panel) or American (middle panel).

*USA–Japan:* After parameterization, the final GBDT model trained on the USA–Japan data consisted of 150 trees, with an interaction depth of 3. The model achieved a moderate accuracy = 0.73 (95% CI [0.70, 0.76]). *USA–China:* After parameterization, the final GBDT model trained on the USA–Japan data consisted of 150 trees, with an interaction depth of 3. The model achieved a moderate accuracy = 0.75 (95% CI [0.72, 0.76]). *Japan–China:* After parameterization, the final GBDT model trained on the USA–Japan data consisted of 150 trees, with an interaction depth of 3. The model achieved a high accuracy = 0.82 (95% CI [0.79, 0.84]). The RVIs for each model are in Table 2, and PDPs for each model are in Figure 2. In short, USA–Japan and USA–China CCMs achieved comparable moderate accuracy, meaning that from the 10 value items in the WVS, sizeable cultural differences were detected. Yet, from the Japan–China model, a higher accuracy score was observed, suggesting that Japan-China differences may be larger than USA–Japan and USA–China differences, at least for the specific set of items used in Analysis 2. In the following section, we unpack the models using RVIs and PDPs to understand where these differences lie.

#### Logistic Regressions

We report the results of the full model followed by the reduced model for each of the cultural comparisons. For all models, due to multicollinearity issues with large numbers of predictors, V79 “To be traditional and religious” had to be excluded to achieve convergence. *USA–Japan.* The full model achieved a fit of McFadden *R*^2^ = 0.15, AIC = 4,680, VIFs range from 1.51 to 2.01; see Table 3, and the reduced model achieved a fit of McFadden *R*^2^ = 0.16, AIC = 4,608. All three variables were significant (V73 “To have a good time”: *b* = −.57, *SE* = 0.04, *Z* = −15.3, *p* < .001, VIF = 1.20; V74 “To do good for society”: *b* = 0.49, *SE* = 0.04, *Z* = 12.1, *p < .*001, VIF = 1.14; V78 “To care for the environment”: *b* = −.56, *SE* = 0.04, *Z* = 14.3, *p <* .001, VIF = 1.20), and the ranking of the effect sizes closely follow the RVI rankings.

**Table 3.** WVS Value Items, (Unstandardized) Coefficients, and *Z*-Scores for Logistic Regressions. Variance Inflation Factors are Available in the Online Supplemental Material.

Items	Label	Value assessed	*b* (*Z*)		
			USA–Japan	USA–China	China–Japan
V70: “It is important to this person to think up new ideas and be creative; to do things one’s own way”	Self-direction	To be creative	0.28*** (6.36)	0.52*** (12.08)	0.90*** (17.34)
V71: “It is important to this person to be rich; to have a lot of money and expensive things”	Power	To be rich	0.48*** (9.54)	−0.92*** (−19.30)	−0.57*** (−10.58)
V72: “Living in secure surroundings is important to this person; to avoid anything that might be dangerous”	Security	To be safe	0.06 (1.21)	0.22*** (4.34)	0.38*** (7.16)
V73: “It is important to this person to have a good time; to ‘spoil’ oneself”	Hedonism	To have a good time	0.73*** (15.65)	0.14** (3.21)	0.87*** (16.89)
V74: “It is important to this person to do something for the good of society”	Benevolence	To do good for society	−0.29*** (−5.63)	0.03 (0.58)	−0.30*** (−4.86)
V75: “Being very successful is important to this person; to have people recognize one’s achievements”	Achievement	To be successful and recognized	0.31*** (6.74)	−0.17*** (−3.83)	0.10 (1.95)
V76: “Adventure and taking risks are important to this person; to have an exciting life”	Stimulation	To be adventurous	−0.12* (−2.56)	0.38*** (8.69)	0.34*** (6.68)
V77 “It is important to this person to always behave properly; to avoid doing anything people would say is wrong”	Conformity	To behave properly	0.19*** (3.77)	0.29*** (5.86)	0.48*** (8.58)
V78: “Looking after the environment is important to this person; to care for nature and save life resources”	Universalism	To care for the environment	0.79*** (15.36)	−0.17*** (−3.47)	0.80*** (12.33)

* *p* < .05. **p < .01. ***p < .001.

##### USA–China

The full model achieved a fit of McFadden *R*^2^ = .22, AIC = 4,591, VIFs range from 1.53 to 2.42; see Table 3, and the reduced model achieved a fit of McFadden *R*^2^ = .19, AIC = 4,746. All three variables were significant (V70 “To be creative”: *b* = .41, *SE* = 0.04, *Z* = 12.1, *p* < .001, VIF = 1.01; V71 “To be rich”: *b* = −1.03, *SE* = 0.04, *Z* = −26.2, *p < .*001, VIF = 1.19; V78 “To care for the environment”: *b* = −.32, *SE* = 0.04, *Z* = −8.29, *p <* .001, VIF = 1.18), and the ranking of the effect sizes closely follow the RVI rankings.

##### Japan–China

The full model achieved a fit of McFadden *R*^2^ = .27, AIC = 4,059, VIFs range from 1.48 to 2.23; see Table 3, and the reduced model achieved a fit of McFadden *R*^2^ = .19, AIC = 4,488. All three variables were significant (V71 “To be rich”: *b* = −.98, *SE* = 0.04, *Z* = −22.6, *p* < .001, VIF = 1.20; V73 “To have a good time”: *b* = .44, *SE* = 0.04, *Z* = 11.9, *p < .*001, VIF = 1.12; V74 “To do good for society”: *b* = −.67, *SE* = 0.05, *Z* = −14.01, *p <* .001, VIF = 1.26), and the ranking of the effect sizes largely follow the RVI rankings.

### Discussion

#### Interpretation of RVIs

While items on values included in WVS (Table 1) are similar to items in Schwartz’s (2006) value questionnaire that are commonly used in cross-cultural analyses, they do not provide a full coverage of Schwarz’s cultural-level circumplex model (Kaasa & Welzel, 2023). For this reason, we interpret each item for the value it assesses. To develop interpretations, our strategy was visual inspections of the PDPs coupled with the RVIs, focusing on value items that contributed most to the classification accuracy of each dyadic cultural model. Specifically, our interpretation focuses on the three items with the highest RVIs that are greater than 10.

With respect to the USA–Japan comparison, the values that contributed most to the classification accuracy were V73 “to have a good time,” V78 “to care for the environment,” and V74 “to do good for society.” The PDP revealed that the stronger the endorsement of V73 “to have a good time” and V78 “to care for the environment,” the higher the likelihood of the respondent being American than Japanese. In contrast, the higher the endorsement of V74 “to do good for society,” the higher the likelihood of the respondent being Japanese than American.

These findings resonate with what is known in the literature on cultural differences between America and Japan. Hedonism, captured in V73 “to have a good time,” is about prioritizing a subjective enjoyment of life and self-indulgence (Schwartz, 2012). Our finding that the valuing of this item differentiates American from Japanese participants resonates with Hofstede’s cultural dimension scores, which list America as higher on indulgence than Japan. Furthermore, past research in culture and affect has also found that Americans prefer the maximization of subjective (hedonic) happiness, whereas happiness in Japan is less positive, and less desirable (Uchida & Kitayama, 2009). To the extent that “to do good for society” captures benevolence and prioritizing of the enhancement and concern for the welfare of the ingroup (Schwartz, 2012), the current finding resonates with the model of cultural differences where Japanese are also more likely to protect social harmony, and also pay more attention to the needs of others in the ingroup as an extension of their self-construal (Markus & Kitayama, 1991). Finally, V78 “to care for the environment” represents universalism in the Schwartz model, which prioritizes the welfare of humankind and the environment in general regardless of ingroup/outgroup status (Schwartz, 2012), which is more strongly endorsed by USA than Japanese respondents. One interpretation could be in cultural differences in prosociality, specifically altruism, between Japan and the USA: Universalism, or the promotion of environmental consciousness, in individualistic, culturally-loose USA, could be an individual’s efforts toward acting in an altruistic and prosocial manner; a reflection of their personal agency in deciding to contribute toward the greater good. Whereas, in collectivistic, culturally-tight Japan, despite more widespread environmentally conscious behaviors, these could be interpreted as conformist attitudes: avoiding social punishment from deviating from environmentally conscious collective norms. This interpretation would be consistent with research showing higher altruism in attitudes toward individual legacies (inheritance; Horioka et al., 2000; Horioka, 2014), and higher prosociality in children (Zahn-Waxler et al., 1996), in the USA than Japan. However, the literature here is sparse, and presents an area of opportunity for future research.

In sum, the USA–Japan model achieved a moderate accuracy score, meaning that sizeable differences exist between U.S. and Japanese respondents, and the three most important variables that were identified by the model in accounting for these differences and are largely consistent with past research.

V70 “To be creative” and V71 “to be rich” underlay the China–U.S. cultural model. The PDP revealed that the stronger the endorsement of V71 “to be rich,” the higher the likelihood of the respondent being Chinese than American, and the higher the endorsement of V70 “To be creative,” the higher the likelihood of the respondent being American than Chinese. Power can be understood as a valuation on wealth and material items for prestige and social status (Schwartz, 2012). Indeed, materialism, as a manifestation of power values, has been well-documented in China (Yang & Stening, 2012), and cross-cultural studies have repeatedly shown it stronger in China than in the USA (e.g., Eastman et al., 1997; Podoshen et al., 2010). One explanation for this could lie in the valuing of collectivistic notions of “face” and reputation coupled with post-Maoist economic reforms that promoted materialism and pragmatism (Yang & Stening, 2012). The high RVI of 40.4 also suggests that, relative to the other predictors, the China–U.S. cultural difference for V71 “to be rich” was especially large. Arguably, “to be creative” may be an expression of the individual’s need for autonomy and independence in everyday life. This is a key feature of individualistic cultures like the USA (Markus & Kitayama, 1991), so the bias toward U.S. classifications for individuals with strong endorsement of this item is unsurprising. In short, the items that contributed most to the classification accuracy of USA–Japan and USA–China comparisons are generally interpretable in terms of past literature.

However, this raises a new question. If self-direction is quintessentially American, why was it not identified in the USA–Japan dyadic model? A look at the PDPs offers some explanation. The direction of classification was consistent across both models; higher self-direction ratings were suggestive of a participant being American rather than Japanese, though the effect on the classification model appeared to be weaker (gentler gradient). In the China–Japan cultural model’s PDP, we see a reason for this weaker effect: Higher self-direction was suggestive of a participant being Japanese than Chinese. In short, this implies that the cultural difference in self-direction between Japan and the USA were not as strong as China and the USA, and this can be further supported by self-direction being more valued in Japan than in China. This is reflected in the RVI list, as RVIs indicate the relative strength of each predictor.

With the USA–Japan and China–USA. CCMs, we establish that our computational approach not only classifies participants accurately, but model interpretations are largely consistent with past research. From this, we can then demonstrate the viability of computational approaches in exploratory research, particularly where there is insufficient theory to determine where and how cultural differences should lie; to “learn” differences between Chinese and Japanese respondents despite the lack of a clear theoretical structure. Our results showed that V71 “to be rich,” V73 “to have a good time,” and V74 “to do good for society” underlay the China–Japan cultural model. The PDP revealed that the higher the rating on V71 “to be rich” and V74 “to do good for society,” the higher the likelihood of the respondent being Chinese than Japanese, but the higher the rating on V73 “to have a good time,” the higher the likelihood of the respondent being Japanese than Chinese. As mentioned earlier, materialism (V71 “to be rich”) is well-documented in research on Chinese society and again serves as an indicator of cultural difference between China and Japan. However, the importance of “having a good time” (V73) in Japan but “doing good for society” (V74) in China may allude to unexplored differences between the two countries in values on self and society, which were previously assumed to be homogeneously Confucian. At the very least, these results suggest that commonly used theories of cultural difference may not be sufficient for explaining these results, and more research is needed to understand cultural variation in values between these countries.

As such, while a detailed commentary on the theoretical implications of these findings is outside the scope of this article, we demonstrate that a data-driven, computational approach can contribute fresh insight into cultural variations within East Asia, and by extension of this approach, other cultural comparisons under-represented in psychological research.

#### Comparisons With Logistic Regressions

Overall, we note that our CCMs in Analysis 2 performed comparably to the logistic regressions but appeared to yield some advantages. Namely, our computational approach seemed better at balancing multicollinearity with model interpretability. For multicollinearity, it becomes unfeasible to fit too many variables in a logistic regression—we were unable to fit all 10 items, and even with nine items the VIF scores were notably higher, and accordingly, the coefficients for each item would not accurately reflect corresponding effect sizes. In contrast, when we repeated the regressions using the top three RVIs (with low VIF scores) as predictor variables in the reduced model, effect sizes largely mirrored their corresponding RVI rankings. This suggests that, when limited to only a handful of strong variables without multicollinearity issues, the RVIs in our CCMs showed high consistencies with easily interpretable traditional logistic regression models. Here, we note that just using 10 items appeared to cause issues with fitting the logistic regression model, whereas machine learning models (such as in Analysis 1) had no issues with fitting 10 times more predictor variables.

Secondly, could model fit be used to measure the magnitude of difference between pairs of countries? We note that the pseudo *R*^2^ scores for the reduced models were largely similar (0.16–0.19), but the pseudo *R*^2^ scores for the full models varied considerably (0.15–0.27) in a similar manner to our CCMs: The Japan–China model fit largely overshadowed the USA–Japan and USA–China fit. This suggests a potential dichotomy: Pseudo *R*^2^ scores seem able to reflect the magnitude of cultural differences in a manner similar to our accuracy scores, when a larger amount of predictors are included in the model. However, this increases the risk of multicollinearity, which makes interpretation difficult. By contrast, small models with a handful of limited, orthogonal items are easy to interpret, but with too few variables, they may not show sufficient differences across pseudo *R*^2^ scores from different dyadic models to represent magnitudes of cultural distance.

Here, our machine learning approach seems able to tackle both issues simultaneously. Moreover, logistic regressions are often used in machine learning, albeit with an introduction of a penalty function to reduce the variance explained by each predictor variable, as a strategy to address the collinearity between predictor variables. These regularized regression models (e.g., ridge) would also balance interpretability and cultural distance estimation and can also be used in place of the GBDT models in CCMs in a similar framework. However, there are pros and cons to this: Our usage of GBDT models additionally accounts for potential interaction effects over simple linear effects but a discussion on the merits of specific machine learning methods is beyond the scope of this article.

## General Discussion

Our analyses showed how interpretable machine learning can be used to obtain estimations of the magnitude of cultural difference between any two cultures. By extrapolating this over a large, cross-cultural dataset, we were able to approximate other measures of cultural distance (CFI) and replicate several key correlations. Compared to CFIs, the main advantage of our model lies in the interpretive power of the dyadic CCMs. For example, we were able to identify the top two RVIs for each cultural model conducted and collate these results for an overview on the items (or domains) that set the USA and China apart. By looking closer at the CCMs and countries for these RVIs, we were able to establish how they differed from specific groups of countries, and these were consistent with past research on American and Chinese demographics and values.

Next, by focusing on USA–Japan, USA–China, and China–Japan comparisons, we showed how our hypothesis-blind computational approach uncovered cultural differences that were previously established and understood for well-studied USA–Japan and USA–China comparisons, as well as novel difference in China–Japan that require further examination. Moreover, at least on values, China–Japan differences appear to be larger than USA–Japan and USA–China differences, suggesting that Chinese and Japanese cultures should not be treated as a homogeneous block for cultural comparisons with the USA, and more research is needed for cultural variation within East Asia.

Where this method could potentially be beneficial could be in cultural comparisons outside WEIRD cultural contexts. Recent movements in cultural psychology have pointed out how existing theories are often overgeneralized. For example, East Asia and Latin America are often grouped as collectivistic and interdependent, and for decades have been treated as similar in cultural comparisons to Anglo-American cultures. However, the rich traditions and philosophies, and ecological affordances give rise to different perspectives and thoughts that are not easily captured in the often *etic*, Western-centric theories used in most cross-cultural research (Krys et al., 2022). However, despite knowing that conventional theories are insufficient, creating new theories of cultural difference for such specialized comparisons would traditionally require time-consuming, *emic*, in-depth observations. Here, we posit that computationally modeling CCMs for indexing cultural differences between several Latin American and East Asian countries would complement (but not replace) these efforts in theory generation through “first look” insights into how these cultures may differ, taking advantage of existing multinational surveys and big data. Given the lack of research in these comparisons, applying CCMs to uncovering, for example, how Brazil differs from China and how Argentina may differ differently from Japan would enrich our understanding of culture on a much more nuanced but global scale.

We also reiterate that while we adopted a nation-level approach to culture, this does not necessarily define real-world cultural boundaries. As an example, a researcher interested in examining cultural differences between island cultures and landlocked cultures could consolidate respondent data from island nations and data from landlocked countries in a similar binary CCM (classifying if respondents are from island or landlocked cultures). Or, researchers interested at looking at different racial or cultural minorities may use similar methods on well-constructed within-country datasets like the New Zealand Attitudes and Values Survey (Sibley, 2023). Essentially, we propose this method as a way to uncover explanations and meaning in theory generation, where large-scale (digital) datasets may exist but before the emergence of theory.

Finally, computational approaches are adept in handling big data. These are not limited to participant responses in surveys as shown in this paper but can also be applied to digital trace data as “cultural products” (see Morling & Lamoreaux, 2008). In recent years, everything that is done online leaves behind behavioral traces or digital footprints. These could be social media posts, search queries, or even the consumption and production of online media. Cultural preferences are often embedded in these data, and computational methods, such as the ones introduced here, can draw insight about cultural mechanisms that influence these patterns of online activity (see Liew et al., 2025).

### Limitations

We note that RVIs and PDPs are interpretative tools designed to understand the “decision-making” processes in machine learning models and cannot substitute for statistical inference. We recommend that researchers who are interested in a particular variable for theoretical reasons also use statistical inference to determine if a predictor is different across cultures. However, for our aims of exploring cultural differences through a bottom-up approach, our priority lies not in hypothesis testing, but in efficiently gaining an overview of the nature of systematic differences between cultures. Secondly, our research relies entirely on the WVS Wave 6. There are some known issues with how data were collected (e.g., Ludeke & Larsen, 2017), and our computational approach necessitates the use of good quality data as a foundation for any kind of inference.

Machine learning models are sensitive to strong patterns in the data. Artifacts, such as missing columns and wrongly coded values for entire groups, could mislead the models into zeroing in on these erroneous entries and falsely inflate accuracy scores. For instance, if one variable was perfectly differentiable between the two countries (e.g., one country had missing data for all the values in that column), the machine learning model would zero into this difference and achieve a perfect differentiation between the two countries. Great care must be given to data cleaning, and observing and removing artifacts, which can be a slow and laborious process depending on the data source. Similarly, this also suggests that variables with strong differences between groups may also hold a larger influence on the distance computations. This could be a potential explanation for the absence of perfect or near-perfect correlations with the CFI, despite using one of the component datasets and similar data-driven approaches. Where the CFIs may potentially “smooth out” specific variables where large differences exist, the machine learning approach may magnify these variables and give them greater weightage during cultural membership classification. Fortunately, this can be accounted for through examining RVIs, and may even be beneficial when working with datasets that are similarly structured and well defined (as in Analysis 2): CCMs become more sensitive in picking up variables that may account for these underlying differences.

## Conclusion

By classifying the culture that a respondent belongs to on the WVS, we show that cultural membership classification using machine learning can be a useful addition to the cultural psychologist’s toolbox. We argue that it excels in exploration and discovery, and with the aid of interpretative tools like the RVI and PDP, can help to uncover novel domains of cultural differences, in cultural comparisons that have been traditionally overlooked by the literature. Furthermore, it can also be used as an interpretable alternative to the CFI, as classification accuracy can also function as an estimate of the magnitude of difference between two cultures. When used appropriately, we think that this can be an exciting method of data exploration, such as the identification of contrasting differences between Japan and China from Analysis 2.

## Supplemental Material

sj-csv-1-psp-10.1177_01461672251339313 – Supplemental material for Machine Learning Culture: Cultural Membership Classification as an Exploratory Approach to Cross-Cultural Psychology Supplemental material, sj-csv-1-psp-10.1177_01461672251339313 for Machine Learning Culture: Cultural Membership Classification as an Exploratory Approach to Cross-Cultural Psychology by Kongmeng Liew, Takeshi Hamamura and Yukiko Uchida in Personality and Social Psychology Bulletin

sj-csv-2-psp-10.1177_01461672251339313 – Supplemental material for Machine Learning Culture: Cultural Membership Classification as an Exploratory Approach to Cross-Cultural Psychology Supplemental material, sj-csv-2-psp-10.1177_01461672251339313 for Machine Learning Culture: Cultural Membership Classification as an Exploratory Approach to Cross-Cultural Psychology by Kongmeng Liew, Takeshi Hamamura and Yukiko Uchida in Personality and Social Psychology Bulletin

## Notes

1.
https://www.oecdbetterlifeindex.org/topics/life-satisfaction/

2. For more information, see https://developers.google.com/machine-learning/crash-course/classification/accuracy

3. For more information, see Finch and Schneider (2006): consistencies between magnitude of group separation (i.e., effect sizes), and classification accuracy across all machine learning algorithms.

4. Data obtained from https://geerthofstede.com

5. This was to minimize cultural differences in response bias. For example, when filling in Likert scales, East Asians generally hovering around the mean, but Americans are more likely to use extremes (Chen et al., 1995).

6. Percentage of population that identify as religiously unaffiliated for Georgia (0.7%), Trinidad and Tobago (1.9%), Zimbabwe (7.9%), and Uzbekistan (0.8%); data from Pew Research Center (2012).

7. Scores for Algeria (−0.78), Egypt (−0.85), Libya (−1.75), Malaysia (−1.38) on the Traditional versus Secular-Rational index (Inglehart & Welzel, 2010): lower scores (negative) indicate higher endorsement of traditional values.
